# Seroprevalence against SARS-CoV-2 after booster vaccination in a prison in Alicante (Spain)

**DOI:** 10.3389/fpubh.2025.1490809

**Published:** 2025-01-28

**Authors:** Ana C. Montagud, Raul Moragues, Nancy Vicente-Alcalde, Emilia Montagud, José Antonio Hurtado-Sánchez, José Tuells

**Affiliations:** ^1^Laboratory of Immunology, Platform of Oncology, Hospital Quironsalud Torrevieja, Alicante, Spain; ^2^Department of Community Nursing, Preventive Medicine and Public Health and History of Science, University of Alicante, Alicante, Spain; ^3^Penitentiary Center Alicante II, Villena, Alicante, Spain; ^4^Department of Nursing, Faculty of Health Sciences, Universidad Cardenal Herrera-CEU, CEU Universities, Elche, Spain; ^5^Primary Care Pharmacy Service, University Hospital of Torrevieja, Alicante, Spain; ^6^Department of Nursing, Faculty of Health Sciences, University of Alicante, Alicante, Spain; ^7^Alicante Institute for Health and Biomedical Research (ISABIAL), Alicante, Spain

**Keywords:** anti-SARS-CoV-2 antibodies, COVID-19, SARS-CoV-2, rapid serological test, lateral flow immunochromatography, prisons

## Abstract

**Background:**

Confinement conditions in prison communities are associated with increased susceptibility to infectious outbreaks. The COVID-19 pandemic has been characterized by high transmissibility and clinical severity resulting in a high number of infections and deaths worldwide. Vaccination has been a crucial tool in mitigating its devastating effects. The aim of this study is to asses the prevalence of antibodies against the Spike protein of SARS-CoV-2 in vaccinated prisoners and staff at a specific prison in Alicante.

**Methods:**

A cross-sectional epidemiological study was designed for the population in scope using a rapid lateral flow immunochromatography serological test, conducted on July 27, 2023. Demographic and clinical variables were collected through a questionnaire. Statistical analysis was performed using the SPSS 29.0 software.

**Results:**

A total of 560 people participated in the study; the predominant profile was men (77.3%) with an average age of 45.7 years. 71.4% of subjects were prisoners and 28.6% were prison staff. Regarding the detection of anti-SARS-CoV-2 antibodies obtained through serological test, 60.9% of the sample gave a positive result. 69.1% of participants received the last dose in 2022 or later and 62.2% received booster doses. The vaccines administered in the last dose were Biontech/Pfizer and Moderna in 88.6% of the cases. 59.5% of sample had suffered from COVID-19 and 67.0% did not have any clinical comorbidity. In the regression analysis, it was observed that the variables with a stronger statistical relationship with presence of anti-SARS-CoV-2 antibodies were: the number of years since last vaccine dose was received (aOR: 0.08; 95%CI: 0.05; 0.16) the number of vaccine doses received (aOR: 4.8; 95%CI: 2.9; 8.0) and presenting any comorbidity (aOR: 4.3; 95%CI: 2.4; 8.0). The staff received more booster doses and obtained a better response to seropositivity, with 72.5% of anti-SARS-CoV-2 result positive while prisoners reached 56.3%.

**Conclusion:**

The COVID-19 vaccination status within the prison community following the initiation of primary immunization and subsequent booster doses, shows a low immunization coverage (60.9%), which is below expectations given the immunization strategies implemented since the start of the pandemic. There are notable differences in vaccination rates between prison staff and prisoners. These disparities are concerning, and authorities responsible for prison public health should take a more proactive approach to ensuring vaccination among prisoners.

## 1 Introduction

The conditions of prisoners in penitentiary centers are associated with greater susceptibility to infectious and transmissible diseases. Prison populations are uniquely vulnerable to infectious diseases due to overcrowding, limited healthcare access, and environmental factors (1). Infection caused by the SARS-CoV-2 virus, characterized by high transmissibility and infectivity, has shown a higher risk of infection in prisons than in general population (2, 3) and even up to five times higher in some countries (4, 5). In Spanish prisons, restrictive measures, such as the suspension of visits and inmate confinement, initially succeeded in controlling transmission, yet outbreaks persisted, reflecting the inherent challenges of managing pandemics in prison settings (6). However, despite the effective implementation of preventive measures, penitentiary centers were not exempt from COVID-19 outbreaks. On January 14, 2021, the first confirmed case was recorded at the Alicante II-Villena penitentiary center (CPAII) following a family visit, leading to an outbreak that affected 10% of the prison population before vaccination had begun (7). This underscores the need for specific preventive measures, such as isolating both symptomatic and asymptomatic individuals, contact tracing, and performing PCR tests (7), in addition to achieving high immunization coverage once a vaccine becomes available.

The vaccine shortage required the prioritization of vaccination for the most vulnerable individuals at risk of contracting COVID-19 to minimize its health and economic impacts. The inclusion of prisoners and prison staff as a high-risk group for vaccination was approached heterogeneously worldwide, with significant differences between countries based on their national policies (8, 9). In Spain, the prison population was included in the COVID-19 vaccination recommendations in March 2021 (10), similar to other countries like Poland and Italy, where high vaccination rates with full schedules were achieved (11–13). Specifically, in Spain, 80% of prisoners had received the full vaccination schedule by June 2021 (6).

In general, vaccination coverage in jails and prisons against other communicable and vaccine-preventable diseases, such as HBV, influenza, MMR and pneumococcus, among others, is low (14, 15). Acceptance of vaccines is considered a significant barrier in penitentiary centers, which may contribute to low vaccination coverage (8, 16). This is often due to distrust in potential side effects, skepticism toward the penitentiary administrative system, lack of information, and a lower perception of the risk of contracting COVID-19 (16–21). In this context, the COVID-19 pandemic has marked a significant shift, driving increased vaccination demand within this group, with a greater interest in the COVID-19 vaccine compared to vaccines for other communicable diseases like influenza (22). At our center, a study conducted in mid-2021 to assess the acceptability of the COVID-19 vaccine found high acceptance rates (23).

In the autumn of 2022, early prevention, monitoring and control strategies for COVID-19 in prisons were updated, and the administration of booster doses to the prison population strongly recommended (24, 25). In Spain, the vaccines used for booster doses were the bivalent vaccines BNT162b2 (Comirnaty®-BioNTech-Pfizer) and mRNA-1273 (Spikevax®-Moderna), both designed to target the original strain and the Omicron variant (25). Published results from the American prison population show favorable outcomes, with a reduction in the risk of COVID-19 infection by 22, 23, and 40% for vaccinated individuals, those with previous infection, and vaccinated individuals with prior infection, respectively (26). These findings highlight a favorable immune scenario resulting from the combination of primary immunization, bivalent booster vaccines, and previous infection, leading to 84% immunity against SARS-CoV-2 in the American prison population (27).

Rapid serological tests based on lateral flow immunochromatography (LFIC) are highly useful tools due to their accessibility, simplicity and cost-effectiveness. They have been employed in numerous epidemiological seroprevalence studies aimed at detecting antibodies against SARS-CoV-2 to assess immune status post-vaccination/infection (28–32).

Given the observed high vaccination acceptance in prison populations and subsequent administration of bivalent booster doses, this study aims to evaluate the humoral immunity resulting from the vaccination using LFIC-based serological tests (32), offering insights into the immune status of this vulnerable population after the implementation of booster doses.

## 2 Materials and methods

### 2.1 Study design

A retrospective cross-sectional epidemiological study was carried out based on the prevalence of antibodies against the “S” protein (Spike) of SARS-CoV-2 in prisoners and staff vaccinated against COVID-19 through a rapid serological test during the month of July 2023 due to availability of resources and the timeline of the vaccination programs. The tests were performed and analyzed by the prison healthcare staff.

### 2.2 Sample

#### 2.2.1 Subjects

The study population consisted of prisoners and prison staff from the CPAII. The inclusion criteria were being an inmate or staff at the Alicante II-Villena prison, over 18 years old, and having been vaccinated with at least one dose of the following vaccines: Comirnaty® (Biontech/Pfizer), Spikevax® (Moderna), Vaxzevria® (AstraZeneca) and Jcovden® (Janssen). All participants were informed about the purpose and implication of the study and voluntarily agreed to participate by signing the informed consent form.

#### 2.2.2 Sample size

Due to a rapidly changing context, we took as baseline the most adverse situation where 50% of the prison population declared having suffered a previous symptomatic COVID-19 infection. Based on this assumption, we designed the study with the goal of estimating the proportion of people with antibodies with 95% confidence and a margin of error of 6%. We assumed a potential type I error rate of 0.05 and of 0.2 for type II error rate. With this configuration, a sample size of 543 was determined.

### 2.3 Detection of antibodies against spike-protein of SARS-CoV-2

“OJABIO® SARS-CoV-2 Neutralizing Antibody Detection Kit (Colloidal Gold Method) from Wenzhou OJA Biotechnology Co., Ltd” was used to detect antibodies against the Spike-protein of SARS-CoV-2. This analytical assay, based on lateral flow immunochromatography was validated using the “surrogate Viral Neutralizing Test” (sVNT) based on “enzyme-linked immunofluorescence assay” (sVNT-ELISA) in healthcare population, which also considered a high-risk group at the time of the study (32). In this test, antibodies against the Spike protein of SARS-CoV-2 present in the sample bind to the SARS-CoV-2 Spike protein conjugated to colloidal gold. The resulting conjugated complex then migrates through the reaction matrix via laminar flow. Once in the reaction matrix, it is captured by anti-Immunoglobulin antibodies (IgG or IgM) fixed to the nitrocellulose membrane. The binding of the antigen–antibody complex is indicated by the formation of a test line (T), signifying the presence of neutralizing antibodies against SARS-CoV-2. An adjacent control line (C) indicates proper technical performance of the test. The presence of both lines is interpreted as a valid positive result. The test readings were performed by two expert researchers following the manufacturer’s instructions.

### 2.4 Variables and statistical analysis

#### 2.4.1 Demographic and clinical variables

The following demographic variables were collected: age, sex, and occupation (prisoner or prison staff). Likewise, epidemiological and clinical variables gathered were: previous infection by SARS-CoV-2, presence of chronic diseases (Chronic Obstructive Pulmonary Disease [COPD], autoimmune diseases, diabetes, high blood pressure), number of doses and type of vaccine received, as well as the year when the person was last vaccinated. For the statistical analysis, the information about chronic diseases was recodified as a binary variable: presence or absence of comorbidity.

#### 2.4.2 Statistical analysis

The data was described using the mean and standard deviation for the continuous variables such as age, vaccine doses received, and years since the last dose. Categorical variables were described using the frequency and proportion of the sample. For both types of variables, 95% Confidence Intervals (CI) were calculated. Furthermore, these were calculated according to the result of the test. Details are broken down for both prison staff and prisoners.

The statistical analysis was performed by calculating Odds Ratios (OR). The OR were calculated by direct calculation for the categorical variables, whereas for the continuous variables logistic regression was used. Furthermore, adjusted Odd Ratios (aOR) were calculated for a specific model including every variable using logistic regression. This model was trained using a stepwise model using Wald’s criterion with a 0.05 significance of entry and 0.10 significance of exit. The data was analyzed using SPSS version 29.0. Results were considered significant when *p* < 0.05.

### 2.5 Ethical considerations

The study was carried out following the bases of the Declaration of Helsinki, and approved by two independent Ethics Committees: the Ethics Committee of the University of Alicante (Spain) (File UA-2021-05-07_5, dated 05/24/2021), and the Ethics Committee of the Health Department of the Dr. Balmis Hospital in Alicante (File PI2021-094, Ref: 2021-0214, dated 06/30/2021). The study was approved by the General Secretariat of Penitentiary Institutions, General Subdirectorate of Institutional Relations and Territorial Coordination (exp. number 74258). The participants were informed about the confidentiality measures and their right to withdraw from the study. The information was treated confidentially, and in accordance with Spanish Organic Law 3/2018, of December 5, on the Protection of Personal Data.

## 3 Results

At the time that the study, the penitentiary center had a total of 896 prisoners and 178 staff members who had received at least one dose of a SARS-CoV-2 vaccine. The entire eligible population was offered the opportunity to participate, voluntarily and anonymously. A total of 560 individuals agreed to participate (400 prisoners and 160 prison staff) resulting in a 52% response rate (560/1,074 total prison population). Among prisoners, the response rate was of 44.6%, while among prison staff, it was 89.9%.

Descriptive statistics of the sample are shown in Table 1. The majority of participants in the study were prisoners (*N* = 400; 71.4%), with the remaining participants being prison staff. Notably, over three-quarters of the participants were male (77.3%). Among prisoners, 89.0% were male whereas in the prison staff, males represented about half of the group. Participants ranged in age from 21 to 65 years old, with an average age of 45.7 years. Both prisoners and prison staff had a similar age distribution, except that there were no prison staff over 60 years of age.

**Table 1:** Description of demographic, clinical and vaccination variables of the study participants (*N* = 560).

N (560)	Total		Antibodies test result		Occupation	
			Negative	Positive	Prisoner	Prison staff
	*Mean (SD)*	*95%CI*	*Mean (SD)*	*95%CI*	*Mean (SD)*	*95%CI*
**Age (years)**	*45.7 (10.3)*	*(44.9; 46.6)*	*44.2 (10.7)*	*46.7 (10.0)*	*45.8 (10.5)*	*45.5 (10.1)*
	***N* (%)**	**95%CI**	***N* (%)**	***N* (%)**	***N* (%)**	***N* (%)**
			**% by row**		**% by column**	
18–29	59 (10.5)	(8.0; 13.1)	34 (57.6)	24 (42.4)	43 (10.8)	16 (10.0)
30–39	99 (17.7)	(14.5; 20.8)	41 (41.4)	58 (58.6)	69 (17.3)	30 (18.8)
40–49	204 (36.4)	(32.4; 40.4)	78 (38.2)	126 (61.8)	143 (35.8)	61 (38.1)
50–59	164 (29.3)	(25.5; 33.1)	50 (30.5)	114 (69.5)	111 (27.8)	53 (33.1)
≥60	34 (6.1)	(4.1; 8.0)	16 (47.1)	18 (52.9)	34 (8.5)	0 (0.0)
**Doses received**	*2.63 (0.86)*	*(2.56; 2.70)*	*1.94 (0.75)*	*3.07 (0.59)*	*2.50 (0.85)*	*2.95 (0.77)*
	***N* (%)**	**95%CI**	***N* (%)**	***N* (%)**	***N* (%)**	***N* (%)**
1 dose	68 (12.1)	(9.4; 14.8)	68 (100.0)	0 (0.0)	59 (14.8)	9 (5.6)
2 doses	144 (25.7)	(22.1; 29.3)	96 (66.7)	48 (33.3)	120 (30.0)	24 (15.0)
3 doses	277 (49.5)	(45.3; 53.6)	55 (19.9)	222 (80.1)	185 (46.3)	92 (57.5)
4 doses	71 (12.7)	(9.9; 15.4)	0 (0.0)	71 (100.0)	36 (9.0)	35 (21.9)
**Years since last dose**	*2.16 (0.66)*	*(2.10; 2.22)*	*2.71 (0.46)*	*1.81 (0.51)*	*2.23 (0.64)*	*2.00 (0.68)*
	***N* (%)**	**95%CI**	***N* (%)**	***N* (%)**	***N* (%)**	***N* (%)**
2021	173 (30.9)	(27.1; 34.7)	155 (89.6)	18 (10.4)	136 (34.0)	37 (23.1)
2022	304 (54.3)	(50.2; 58.4)	64 (21.1)	240 (78.9)	218 (54.5)	86 (53.8)
2023	83 (14.8)	(11.9; 17.8)	0 (0.0)	83 (100.0)	46 (11.5)	37 (23.1)
	***N* (%)**	**95%CI**	***N* (%)**	***N* (%)**	***N* (%)**	***N* (%)**
**Test result**			**% by row**		**% by column**	
Negative	219 (39.1)	(35.1; 43.1)				
Positive	341 (60.9)	(56.9; 64.9)				
**Occupation**						
Prisoner	400 (71.4)	(67.7; 75.2)	175 (43.8)	225 (56.3)		
Prison staff	160 (28.6)	(24.8; 32.3)	44 (27.5)	116 (72.5)		
**Sex**						
Male	433 (77.3)	(73.9; 80.8)	173 (40.0)	260 (60.0)	356 (89.0)	77 (48.1)
Female	127 (22.7)	(19.2; 26.1)	46 (36.2)	81 (63.8)	44 (11.0)	83 (51.9)
**Previous SARS-CoV-2 infection**						
No	227 (40.5)	(36.5; 44.6)	100 (44.1)	127 (55.9)	181 (45.3)	46 (28.7)
Yes	333 (59.5)	(55.4; 63.5)	119 (35.7)	214 (64.3)	219 (54.8)	114 (71.3)
**Chronic disease**						
Autoimmune	39 (7.0)	(4.9; 9.1)	0 (0.0)	39 (100.0)	37 (9.3)	2 (1.3)
Diabetes	43 (7.7)	(5.5; 9.9)	3 (7.0)	40 (93.0)	40 (10.0)	3 (1.9)
COPD	19 (3.4)	(1.9; 4.9)	0 (0.0)	19 (100.0)	19 (4.8)	0 (0.0)
High Blood Pressure	84 (15.0)	(12.0; 18.0)	25 (29.8)	59 (70.2)	78 (19.5)	6 (3.8)
None	375 (67.0)	(63.1; 70.9)	191 (50.9)	184 (49.1)	226 (56.5)	149 (93.1)
**Vaccine administered in the last dose received**						
AstraZeneca	3 (0.5)	(0.0; 1.1)	0 (0.0)	3 (100.0)	0 (0.0)	3 (1.9)
Biontech/Pfizer	312 (55.7)	(51.6; 59.8)	109 (34.9)	203 (65.1)	209 (52.3)	103 (64.4)
Janssen	61 (10.9)	(8.3; 13.5)	61 (100.0)	0 (0.0)	59 (14.8)	2 (1.3)
Moderna	184 (32.9)	(29.0; 36.7)	49 (26.6)	135 (73.4)	132 (33.0)	52 (32.5)

Breakdown according to the antibodies test result and the prisoner or prison staff status of participants.

SD, Standard deviation; CI, Confidence Interval; COPD, Chronic obstructive pulmonary disease.

Out of the 560 tests performed, 341 (60.9%) yielded a positive result, showing the presence of antibodies against the Spike-protein of SARS-CoV-2. The positivity was higher among prison staff (72.5%) than among prisoners (56.3%). There were no significant differences in positivity anti-SARS-CoV-2 antibodies according to gender, with 60.0% positivity in men and 63.8% in women (*p* = 0.448). The average age of those who tested positive was slightly higher than among those who tested negative (2.5 years older, *p* = 0.004).

Every participant in the study was vaccinated with at least one vaccine dose. Approximately one half of the subjects declared having received 3 vaccine doses, while a quarter of the sample had received 2 doses, and the remaining subjects were split similarly between 1 and 4 vaccine doses. Those who tested positive had received more vaccine doses, with 3.07 ± 0.59 vaccine doses on average, than those who obtained a negative test result, who had received 1.94 ± 0.75 doses. Prison staff had received more vaccine doses (2.95 ± 0.77) than prisoners, who had received 2.50 ± 0.85 doses.

While 173 (30.9%) participants had not been vaccinated since 2021, slightly over half participants were vaccinated for the last time in 2022, and the remaining 15% had been vaccinated in 2023. The time elapsed since the last vaccination dose was higher among those who tested negative (2.71 ± 0.46 years) than those with a positive antibody test result (1.81 ± 0.51 years), and higher among prisoners (2.23 ± 0.64 years) than within prison staff (2.00 ± 0.68 years).

Six out of 10 participants declared having suffered a previous SARS-CoV-2 infection. Those who had suffered a previous infection showed a significantly higher positivity (64.3%) than those who had not (55.9%) with a *p*-value for the chi-squared test with 1 degree of freedom of *p* = 0.048 which indicates that this difference can be considered as statistically significant at a significance level of *α* = 0.05. Additionally, the incidence of SARS-CoV-2 infection was higher among prison staff (71.3%) than prisoners (54.8%). Around two thirds of the subjects who had suffered a prior infection had antibodies. This was similar in prisoners (63.5%) and prison staff (65.8%), but the positivity was different among those who had not had a previous infection. While 89.1% of the prison staff without a previous infection presented a positive test result, slightly below half of the prisoners who had not suffered a previous SARS-CoV-2 infection showed presence of antibodies (47.5%).

Finally, around two out of every three subjects declared having no chronic diseases. Among those who had some comorbidity, 174 were prisoners and 11 were prison staff, indicating a presence of comorbidity in 43.5% of prisoners and only in 6.9% of prison staff. The chronic diseases present were distributed with 15% of subjects reporting high blood pressure, around 7% declaring having an autoimmune disease or diabetes, while the remaining 3.4% suffered from chronic obstructive pulmonary disease. Regarding positivity and comorbidities, prisoners with comorbidity had a positivity of 89.1% compared to prisoners without comorbidities where 31.0% presented antibodies. Furthermore, prisoners with comorbidity have received more vaccine doses (2.99 vs. 2.12 doses); more recently (1.86 years vs. 2.51 years) and are older (49.0 vs. 43.4 years) than prisoners without comorbidities. We would like to highlight how the results obtained for the prison staff differ significantly as we can see next. Those participants without comorbidities obtained a higher positivity (76.5% vs. 18.2%), with no significant differences observed between age or the number of doses received. On the contrary, the variable time since the last dose is influential, since prison staff without comorbidities have received vaccines more recently (1.94 vs. 2.82 years). Regarding the comparison between participants with comorbidities, according to whether they are prisoners or staff, we observe that prisoners with comorbidities have received more vaccine doses (2.99 vs. 2.54 doses), more recently (1.86 vs. 2.82 years ago) and show a higher positivity of 89.1%, which contrasts with the 18.2% among staff with comorbidities. However, there were only 11 members of the prison staff with comorbidities in the sample, which requires non-parametric tests for comparison. The *p*-values for the Mann–Whitney U test for the quantitative variables and of Fisher’s exact test for positivity were significant in all comparisons indicated (*p* < 0.001).

Regarding the vaccines administrated in the last administered dose, 88.6% of the subjects received the Biontech/Pfizer and Moderna vaccines. The positivity rate for these subjects was greater than 65%. Those subjects (59 prisoners and 2 prison staff) who received the last dose of the Janssen vaccine in 2021, present a 100% antibody negativity rate. In contrast, 3 out of 3 of the subjects (prison staff) who received the last dose of the AstraZeneca vaccine presented a positive serological result in antibodies although the small sample size of three subjects makes unadvisable drawing strong conclusions.

To assess the statistical significance of these differences in seroprevalence, we calculated the OR for each variable, which can be found in Table 2. When analyzed separately, we observe that all variables considered except for the gender of a participant show significant differences in risk. In particular, the proportion of positive test results was higher in older people, and among those with comorbidities. The positivity was also higher in those who had received more vaccine doses, in those vaccinated more recently, and among those who had previously suffered a SARS-CoV-2 infection.

**Table 2:** Positivity in the antibody test according to demographic, clinical and vaccination variables.

Variable	Reference	OR	95%CI	aOR	95%CI
Age (decades)	–	1.27*	(1.08; 1.50)	n.s.	
Vaccine doses received^*^	–	11.14*	(7.57; 16:40)	4.8	(2.9; 8.0)
Years since last dose^*^	–	0.029*	(0.017; 0.05)	0.08	(0.05; 0.16)
Occupation	Prisoner	2.05*	(1.38; 3.06)	n.s.	
Sex	Male	1.17	(0.78; 1.77)	n.s.	
Previous SARS-CoV-2 infection	No	1.42*	(1.003; 1.999)	n.s.	
Chronic disease^*^	No	5.82*	(3.71; 9.13)	4.3	(2.4; 8.0)

OR of each variable with the result of the test and aOR corresponding to the multivariate logistic regression model.

* Significant *p* < 0.05; n.s., non-significant; OR, Odds Ratio; aOR, adjusted Odds Ratio; CI, Confidence Interval.

Additionally, a multivariate analysis was performed using logistic regression to adjust for confounding variables. The result of the test was used as target variable and all the remaining variables were used as co-variables, with comorbidity in their binary recodification. The variables age (in decades), vaccine doses received and years since the last dose were included as continuous variables. This model was used to calculate the adjusted aOR. The variables that yielded a significantly higher proportion of positive test results were: having received more doses, i.e., each additional dose increases the odds of a positive test by aOR: 4.8; 95%CI: (2.9; 8.0); lower time since the last vaccine dose, i.e., each year longer since the last vaccination reducing the odds of presenting antibodies by aOR: 0.08; 95%CI: (0.05; 0.16); and suffering a comorbidity (aOR: 4.3; 95%CI: 2.4; 8.0). The remaining variables did not appear as statistically significant in this model.

Regarding potential collinearity between variables, an analysis of the predictive variables yielded variance inflation factors (VIF) with values smaller than 1.6 for all variables except for the number of doses (VIF = 2.86) and the years since the last dose (VIF = 2.57), which are within acceptable levels. Therefore, there is little indication of multicollinearity causing problems in this model.

## 4 Discussion

Available seroepidemiological data on SARS-CoV-2 in prison settings are limited. Reported immunization coverage against SARS-CoV-2 in prisons varies significantly between countries and is influenced by the timing of the pandemic and vaccination policies (33–36). This study is the first seroprevalence study conducted in a penitentiary institution in Spain following the onset of the COVID-19 pandemic and the implementation of booster doses.

The most efficient vaccination strategy reported in prisons against SARS-CoV-2 is the immunization of inmates and staff over 50 years of age, which leads to a significant reduction in COVID-19 incidence by more than 50%, as well as a 41.1% reduction in cases and a 35.9% reduction in deaths (37). In this study, the participants were predominantly young men, with the average age being below 50 years for both prisoners and prison staff. Overall, 60.9% of the participants had antibodies against the Spike protein of SARS-CoV-2, as detected by a rapid serological test, following the implementation of booster doses. There was a trend toward greater positivity with increasing age, regardless of gender.

The presence of anti-SARS-CoV-2 antibodies is influenced by several factors, including previous infection, the time elapsed since the last exposure to the virus (whether through infection or vaccination), and the number of vaccine doses received (28–31, 38). In the sample, the results confirm that having had the disease predisposes individuals to the presence of antibodies, regardless of occupation. Paradoxically, there were significant differences between prisoners and prison staff who had not been infected, with the latter group showing higher positivity for anti-SARS-CoV-2 antibodies. Additionally, the administration of booster doses was associated with much higher positivity for Spike-protein anti-SARS-CoV-2 antibodies. Specifically, it is noteworthy that prison staff received more booster doses and had a better response to seropositivity, with 72.5% positivity compared to 56.3% among prisoners. Given that prison staff received more vaccine doses, this suggests that the presence of antibodies in those who have not been infected with COVID-19 depends on the number of vaccine doses received, indicating that the immunization achieved is a result of the booster vaccination.

**Figure 1:**
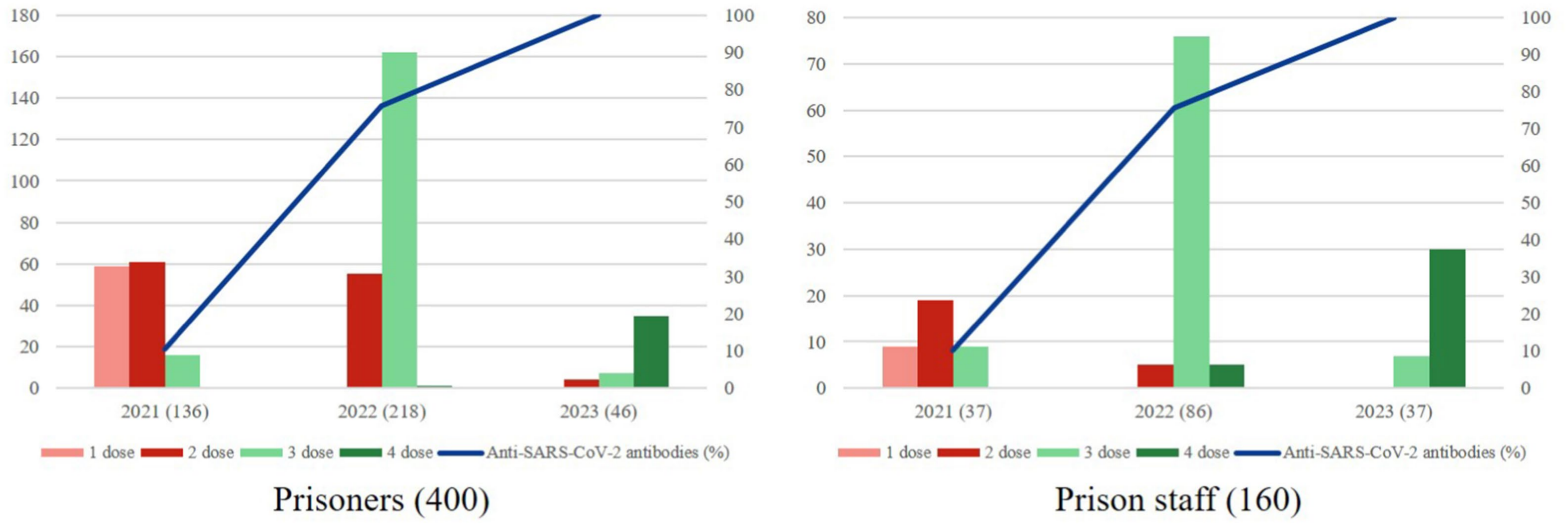
Relation among the distribution of sample according to the year of the last dose vaccine administered and percentage of anti-SARS-CoV-2 antibodies on prisoners and prison staff. Both graphs show each occupational group stratified by the last year in which they were administered a vaccine dose. The left axis shows the number of patients (n) who had received 1, 2, 3, or 4 doses in the last vaccine administration. The right axis shows the percentage of subjects with anti-SARS-CoV-2 antibodies according to the year of the last dose vaccine.

Additionally, a significant difference in the presence of antibodies was observed based on the time elapsed since the last dose was administered. The positivity rate for participants vaccinated within the last year (2023) was 100%, in contrast to those vaccinated two or more years ago, who had a lower positivity rate. However, booster doses were first administered in 2022, and the positivity rate for those vaccinated in that year reached almost 80% (Figure 1). This suggests that the immunization coverage achieved after booster doses is maintained even 2 years after the last dose. In other words, the number of years since the last dose may act as a protective factor.

Most participants were vaccinated in 2022, but differences were observed between prisoners and prison staff in the last year, with a lower vaccination rate among the former (Figure 1). According to data published by Vicente-Alcalde et al., vaccine acceptance during the initial immunization period was 90% among prisoners (23), suggesting that a higher vaccination rate could be expected. However, in this study, the response rate among prisoners (44,6%) was low, which could be considered a limitation. Furthermore, the high turnover in this group makes it challenging to monitor vaccination programs, as it is difficult to track vaccinations continuously, ensure that everyone receives the necessary doses, and maintain accurate records, as prisoners and staff enter and leave the system. Likewise, the decrease in the incidence and severity of COVID-19, combined with the voluntary nature of vaccination after primary immunization, may have contributed to a lower perception of risk among prisoners, which is reflected in the lower vaccination rates.

Regarding comorbidities, 94% of the subjects affected by them were prisoners who had received the vaccine, despite their status as a vulnerable group due to social, hygienic, confinement, or institutional health dependency factors. We observed that 86.5% of subjects with comorbidities had received a vaccine dose in 2022 or later, compared to only 60.5% of those without comorbidities. This suggests a high level of awareness and vaccine acceptance among prisoners with chronic diseases, as well as effective management of vaccination programs for prisoners with comorbidities by prison healthcare staff.

The two main vaccines administered at CPAII are mRNA-based, and both have achieved a positivity rate for anti-SARS-CoV-2 antibodies greater than 65%. These vaccines are designed to induce a specific immune response against the SARS-CoV-2 Spike protein, resulting in the formation of neutralizing antibodies (39). However, not all anti-SARS-CoV-2 Spike antibodies exhibit neutralizing activity, nor do they have the same efficacy against all variants (30–42). Based on published data, it appears that additional vaccine doses enhance neutralization (41). Stufano et al. observed that the neutralizing capacity of antibodies generated after the initial vaccination schedule was limited against the Omicron variant, but this capacity increased when booster doses of the BNT162b2 mRNA vaccine were administered (38). These findings support the need to continue vaccination programs with booster doses of mRNA vaccines in prison settings to ensure maximum protection and safety against potential critical variants of SARS-CoV-2.

Among the limitations of the study, the overall response rate of 52% should be highlighted. However, when considering participation by occupational group, we observed a higher response rate among prison staff (89.9%) compared to prisoners (44.6%). Additionally, while neutralizing antibodies are those that confer immunity, the serological test used in this study does not specifically evaluate the neutralizing capacity of Spike-protein antibodies. Nevertheless, it has been previously validated for detecting neutralizing antibodies against SARS-CoV-2 (32). Moreover, comparative studies between serological techniques and neutralization assays have shown a high correlation in their results (32, 43). These findings support the use of rapid serological tests as a useful tool for assessing the humoral immunity generated against SARS-CoV-2 in high-risk environments, such as the prison population. Additionally, while sex and age were considered as variables, there could be other potential confounders such as socio-demographic differences between prisoners and staff. Other socio-demographic variables, such as the prisoners’ employment prior to incarceration, place of birth, education level, marital status, etc., were not collected and, therefore, were not included in the analysis.

In conclusion, the findings indicate relatively low vaccination coverage against COVID-19 among prisoners compared to prison staff, underscoring the need to continue booster vaccination programs to achieve sustained immunity against SARS-CoV-2, as supported by other studies (38–42). Rapid serological tests can serve as a valuable tool for guiding preventive policies against communicable diseases such as COVID-19 in prison settings.

## Data Availability

The raw data supporting the conclusions of this article will be made available by the authors without undue reservation.
